# The risk of smoking on multiple sclerosis: a meta-analysis based on 20,626 cases from case-control and cohort studies

**DOI:** 10.7717/peerj.1797

**Published:** 2016-03-15

**Authors:** Peng Zhang, Rui Wang, Zhijun Li, Yuhan Wang, Chunshi Gao, Xin Lv, Yuanyuan Song, Bo Li

**Affiliations:** Department of Epidemiology and Biostatistics, Jilin University School of Public Health, Changchun, China

**Keywords:** Meta-analysis, Multiple sclerosis, Smoking

## Abstract

**Background.** Multiple sclerosis (MS) has become a disease that represents a tremendous burden on patients, families, and societies. The exact etiology of MS is still unclear, but it is believed that a combination of genetic and environmental factors contribute to this disease. Although some meta-analyses on the association between smoking and MS have been previously published, a number of new studies with larger population data have published since then. Consequently, these additional critical articles need to be taken into consideration.

**Method.** We reviewed articles by searching in PubMed and EMBASE. Both conservative and non-conservative models were used to investigate the association between smoking and the susceptibility to MS. We also explored the effect of smoking on the susceptibility to MS in strata of different genders and smoking habits. The association between passive smoking and MS was also explored.

**Results.**The results of this study suggest that smoking is a risk factor for MS (conservative model: odds ratio (OR) 1.55, 95% CI [1.48–1.62], *p* < 0.001; non-conservative model: 1.57, 95% CI [1.50–1.64], *p* < 0.001). Smoking appears to increase the risk of MS more in men than in women and in current smokers more than in past smokers. People who exposed to passive smoking have higher risk of MS than those unexposed.

**Conclusion.**This study demonstrated that exposure to smoking is an important risk factor for MS. People will benefit from smoking cessation, and policymakers should pay attention to the association between smoking and MS.

## Introduction

Multiple sclerosis (MS) is an inflammatory disease that occurs when the spinal cord and the insulating covers of the nerve cells in the brain are damaged. This damage affects the nervous system’s ability to communicate, resulting in a number of physical and mental problems (Compston & Coles, 2002; Compston & Coles, 2008). Evidence indicates that MS is an autoimmune disease that directly affects the central nervous system (CNS) myelin or oligodendrocytes. A variety of neurological signs and symptoms are determined by the distribution of white matter lesions in the nervous system that may occur in sudden attacks or build up over time (Compston & Coles, 2008).

In 2013, there were about 1.5 million people who suffered from MS around the world, with rates varying widely in different regions and populations (WHO, 2013). 19,800 people died from MS in 2013, a statistic that was up from 12,400 people in 1990 (Collaborators, 2015). The disease usually occurs between the ages of 20 and 50, occupying the leading position of disability among young adults. The risk of MS for females is two times as high as males (Milo & Kahana, 2010).

The cause of MS is still not clear, but through rigorous epidemiological investigation, genetic variations, the Epstein–Barr virus infection, vitamin D nutrition and cigarette smoking have been identified as likely causal factors for MS (Handel et al., 2010; Ramagopalan et al., 2009; Simon et al., 2012).

A previous meta-analysis published in 2014 reported a pooled odds ratio (OR) of 1.51 (95% CI [1.38–1.65]) for the association between smoking and MS susceptibility (O’Gorman & Broadley, 2014). However, the evidence was suggestive rather than sufficient about the role of smoking in the etiology of MS because the sample sizes were relatively small. Many recent studies have explored the association between smoking and MS either directly or indirectly. Therefore, we conducted this meta-analysis to investigate the association in a larger sample. Moreover, we aimed to detect the effect of smoking on the incidence of MS in strata of different genders and smoking habits.

## Materials and Methods

### Search strategy

We identified published studies that explored the association between smoking and the risk of MS by searching the PubMed and EMBASE databases from January 1st, 1980 to March 31st, 2015. The following search terms were used: “multiple sclerosis,” “case-control,” “cohort study,” “birth cohort,” “survival analysis,” “cigarette smoking,” “tobacco smoking” and “cigars.” In addition, the reference list of retrieved papers was also reviewed to identify additional relevant studies.

### Selection criteria

The eligible studies needed to meet the following criteria: (1) the study must be an original study, (2) the study must investigate the association between smoking and the incidence of multiple sclerosis, (3) the study must include at least 50 cases, and (4) the study must report the odds ratio (OR), relative risk (RR) with its corresponding 95% confidence interval (95% CI), or the number of events to calculate them.

### Study selection and data extraction

The articles retrieved from the database were independently evaluated by two reviewers (Peng Zhang and Rui Wang) based on the aforementioned selection criteria. Studies designed as systematic review and duplicate studies of the same population were excluded. Articles that contained multiple study populations were divided into separate studies. Disagreements were resolved by discussion. Articles in which disagreements could not be resolved were all included. The following information were extracted from the eligible studies: first author, year of publication, country of origin, OR or RR with its 95% CI, study design, the method of information collection, method of MS diagnosis, and the relationship between disease onset and the duration of smoking.

### Statistical analysis

The rare disease assumption was used to combine the odds ratio (OR) and relative risk (RR) (Clayton & Hills, 1993). If the RR or OR and its 95% CI were not reported but sufficient information was available, we used previously described methods to calculate it (Bland & Altman, 2000). Stata12.0 was used to compute the pooled ORs and their 95% CI, to generate forest plots and to assess the heterogeneity of the included studies. As described in the former meta-analysis (Handel et al., 2011), we also performed this meta-analysis using conservative (including only studies where smoking behavior was described prior to disease onset) and non-conservative (all studies regardless of whether smoking behavior occurred before onset or concurrently) models. To test the stability of the results, we investigated the influence of a single study on the overall effect value by removing one study each time. ORs were calculated among the subgroups of studies and compared across them. Possible publication bias was assessed using Begg’s funnel plot and Egger’s test (Begg & Mazumdar, 1994; Egger et al., 1997).

## Results

### Search result and study characteristics

**Figure 1:**
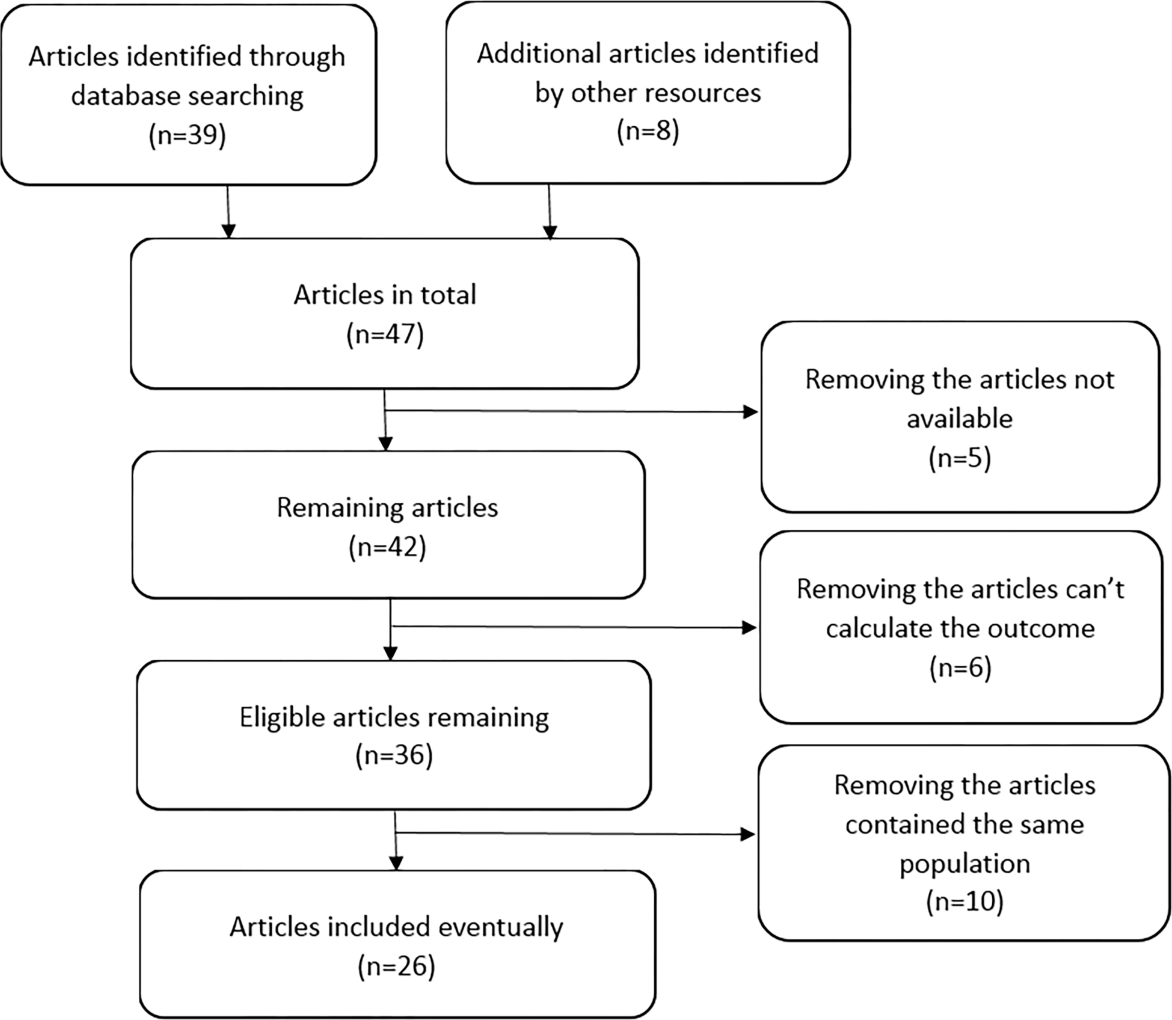
Summary of the studies selection process.

After selecting studies according to the inclusion criteria, 47 articles considered for further review. Six of these 47 articles could not provide outcome information (Brosseau et al., 1993; Guimond et al., 2014; Lauer, 2006; Nortvedt, Riise & Maeland, 2005; Senecal-Quevillon, Duquette & Richer, 1986; Turner et al., 2007). We could not obtain the full article for 5 of the 47 articles (Dobosz, Tyrpien & Pierzchala, 2012; Frutos Alegria et al., 2002a; Frutos Alegria et al., 2002b; Ragonese et al., 2007; Rodriguez Regal et al., 2009). Ten of these 47 articles contained duplicate study populations (Baarnhielm et al., 2012; Hedstrom et al., 2011a; Hedstrom et al., 2009; Hedstrom et al., 2013a; Hedstrom et al., 2014b; Hedstrom et al., 2011b; Munger, Chitnis & Ascherio, 2009; Munger et al., 2003; Sundqvist et al., 2012; Sundstrom, Nystrom & Hallmans, 2008). Ultimately, 26 eligible articles containing 29 study populations were identified (Al-Afasy et al., 2013; Alonso et al., 2011; Asadollahi et al., 2013; Briggs et al., 2014; Carlens et al., 2010; Ghadirian et al., 2001; Gustavsen et al., 2014; Hedstrom et al., 2013b; Hernan et al., 2005; Hernan, Olek & Ascherio, 2001; Jafari et al., 2009; Kotzamani et al., 2012; Maghzi et al., 2011; Mansouri et al., 2014; O’Gorman et al., 2014; Pekmezovic et al., 2006; Ragnedda et al., 2015; Ramagopalan et al., 2013; Riise, Nortvedt & Ascherio, 2003; Russo et al., 2008; Silva et al., 2009; Simon et al., 2015; Simon et al., 2010; Thorogood & Hannaford, 1998; Villard-Mackintosh & Vessey, 1993; Zorzon et al., 2003). A flow chart for the study selection process was shown in Fig. 1. There were 19,834 cases of MS and 21,350 controls in case-control studies; 792 cases of MS occurred in 601,492 individuals in cohort studies. Among these studies, four were conducted in Iran, four in America, three in England, three in Norway, two in Canada, three in Sweden, one in Brazil, one in Greece, two in Australia, one in the Netherlands, one in Kuwait, three in Italy, one in Serbia. The main characteristics of the included studies are summarized in Table 1.

**Table 1:** The main characteristics of the included studies.

1st author and year of publication	Cases	Controls or observational individual	OR or RR(95% CI) versus never-smoking	Information collecting	Type	Diagnostic criteria	Smoking and the onset of MS
Ragnedda 2015 (Norwegian)	894	1,610	2.00(1.68, 2.38) (ever-smoking)	Questionnaire	Case-control	McDonald	Before onset
Ragnedda 2015 (Italian)	617	1,161	1.55(1.28, 1.88) (ever-smoking)	Questionnaire	Case-control	McDonald	Before onset
Simon 2014	1,190	454	1.4(1.1, 1.9) (ever-smoking)	Face interview	Case-control	N/A	Before onset
Gustavsen 2014	530	918	2.29(1.82, 2.89) (ever-smoking)	Questionnaire	Case-control	McDonald or Poser	current
Mansouri 2014	1,217	787	1.93(1.31, 2.73) (ever-smoking)	Face interview	Case-control	McDonald or Poser	Before onset
O’Gorman 2014	560	480	1.9(1.5, 2.5) (ever-smoking) 3.6(2.3, 5.6) (current-smoking) 1.6(1.2, 2.1) (past-smoking)	Questionnaire	Case-control	Physician	Current
Briggs 2014	1,012	576	1.27(1.03, 1.58) (ever-smoking)	Telephone questionnaire	Case-control	McDonald	Before onset
Asadollahi 2013	662	394	1.78(1.22, 2.59) (ever-smoking)	Face or telephone interview	Case-control	McDonald or Poser	Before onset
Hedström 2013	6,990	8,279	1.49(1.40, 1.59) (ever-smoking) 1.56(1.45, 1.67) (current-smoking) 1.35(1.24, 1.47) (past-smoking)	Questionnaire	Case-control	McDonald	Before onset
Ramagopalan 2013	3,157	756	1.32(1.10, 1.60) (ever-smoking)	Questionnaire	Case-control	N/A	Current
Kotzamani 2012	504	591	1.9(1.50, 2.41) (ever-smoking)	Questionnaire	Case-control	N/A	Before onset
Al-Afasy 2010	101	202	1.7(0.9, 2.4) (ever-smoking)	Face interview	Case-control	Neurologist	Before onset
Maghzi 2011	516	1,090	2.67(1.70, 4.21) (ever-smoking)	Questionnaire	Case-control	McDonald	Before onset
Alonso 2011	394	394	1.72(0.90, 3.30) (ever-smoking)	Telephone interview	Case-control	McDonald	Before onset
Simon 2010a	210	420	1.4(1.0, 2.0) (ever-smoking)	Questionnaire	Case-control	N/A	Before onset
Simon 2010b	136	272	1.5(1.0, 2.4) (ever-smoking)	Interview	Case-control	Poser	Before onset
Simon 2010c	96	173	1.4(0.8, 2.4) (ever-smoking)	Questionnaire	Case-control	N/A	Before onset
Carlens 2010	214	277,777	2.5(1.7, 3.6) (ever-smoking) 2.8(1.9, 4.2) (current-smoking) 1.6(0.9, 2.8) (past-smoking)	N/A	Cohort	N/A	Before onset
Jafari 2009	136	204	1.09(0.68, 1.73) (ever-smoking) 1.03(0.61, 1.73) (current-smoking) 1.19(0.65, 2.20) (past-smoking)	Questionnaire	Case-control	McDonald	Before onset
Silva 2009	81	81	2.0(0.9, 4.3) (current-smoking)	Face interview	Case-control	Poser	Current
Russo 2008	94	53	1.83(0.86, 3.87) (ever-smoking)	N/A	Case-control	McDonald	N/A
Pekmezovic 2006	196	210	1.6(1.08, 2.37) (ever-smoking)	Face interview	Case-control	Poser	Before onset
Hernan 2005	210	1,913	1.3(1.0, 1.7) (ever-smoking) 1.4(1.0, 1.9) (current-smoking) 1.0(0.6, 1.8) (past-smoking)	Questionnaire	Case-control	Poser	Before onset
Riise 2003	87	22,312	1.81(1.13, 2.92) (ever-smoking)	Questionnaire	Cohort	Self-report	Before onset
Zorzon 2003	140	131	1.50(0.90, 2.40) (ever-smoking)	Interview	Case-control	McDonald	Before onset
Hernan 2001	314	238,371	1.6(1.2, 2.1) (current-smoking) 1.2(0.9,1.6) (past-smoking)	Questionnaire	Cohort	Physician	Before onset
Ghadirian 2001	200	202	1.6(1.0, 2.4) (ever-smoking)	Face interview	Case-control	N/A	Before onset
Thorogood 1998	114	46,000	1.2(0.8, 1.8) (1–14/day)	N/A	Cohort	Physician	Before onset
Villard 1993	63	17,032	1.5(0.6, 3.3) (ever-smoking)	N/A	Cohort	N/A	Before onset

### Smoking and MS susceptibility

**Figure 2:**
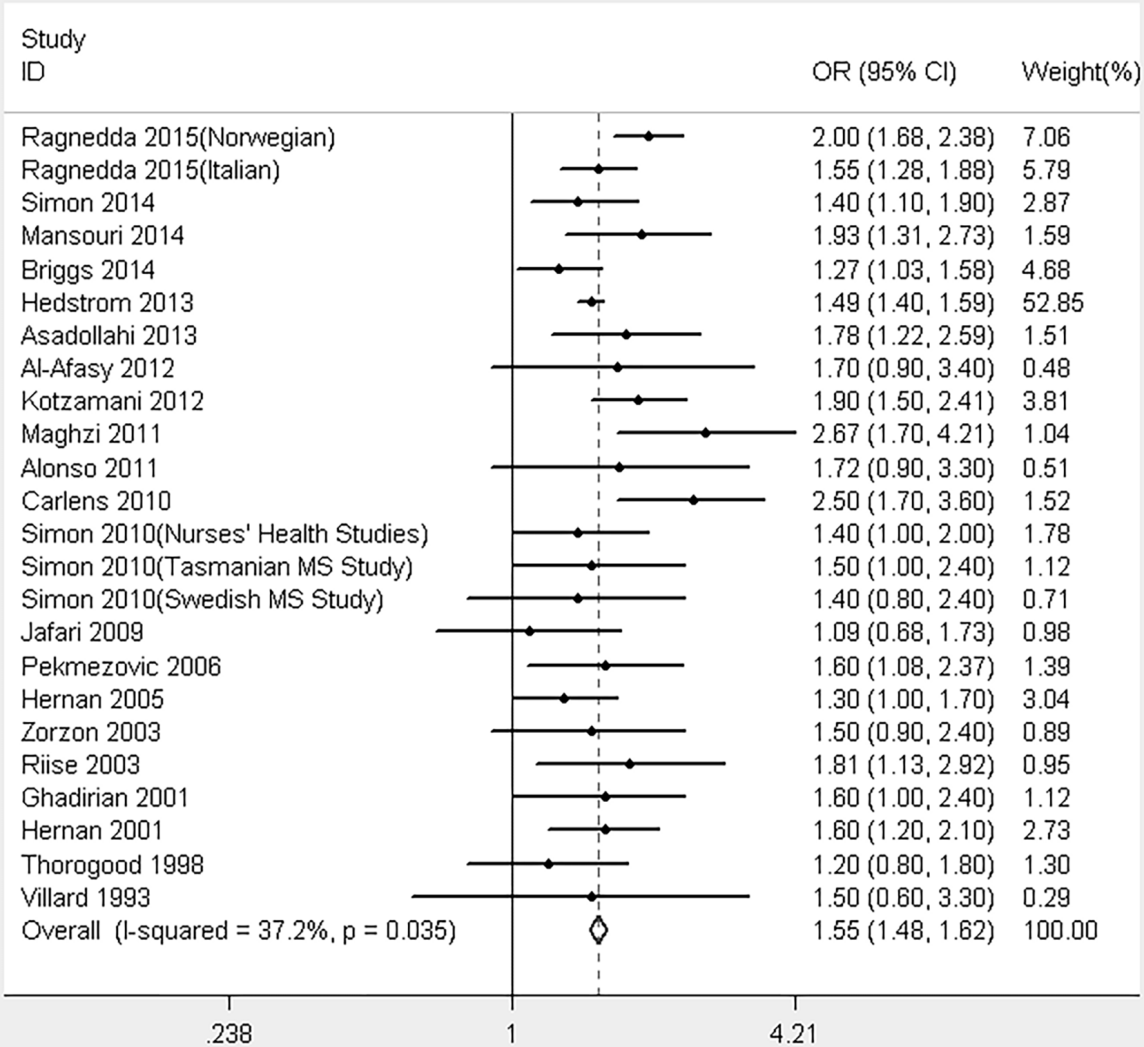
Forest plot of smoking and multiple sclerosis risk (conservative model).

**Figure 3:**
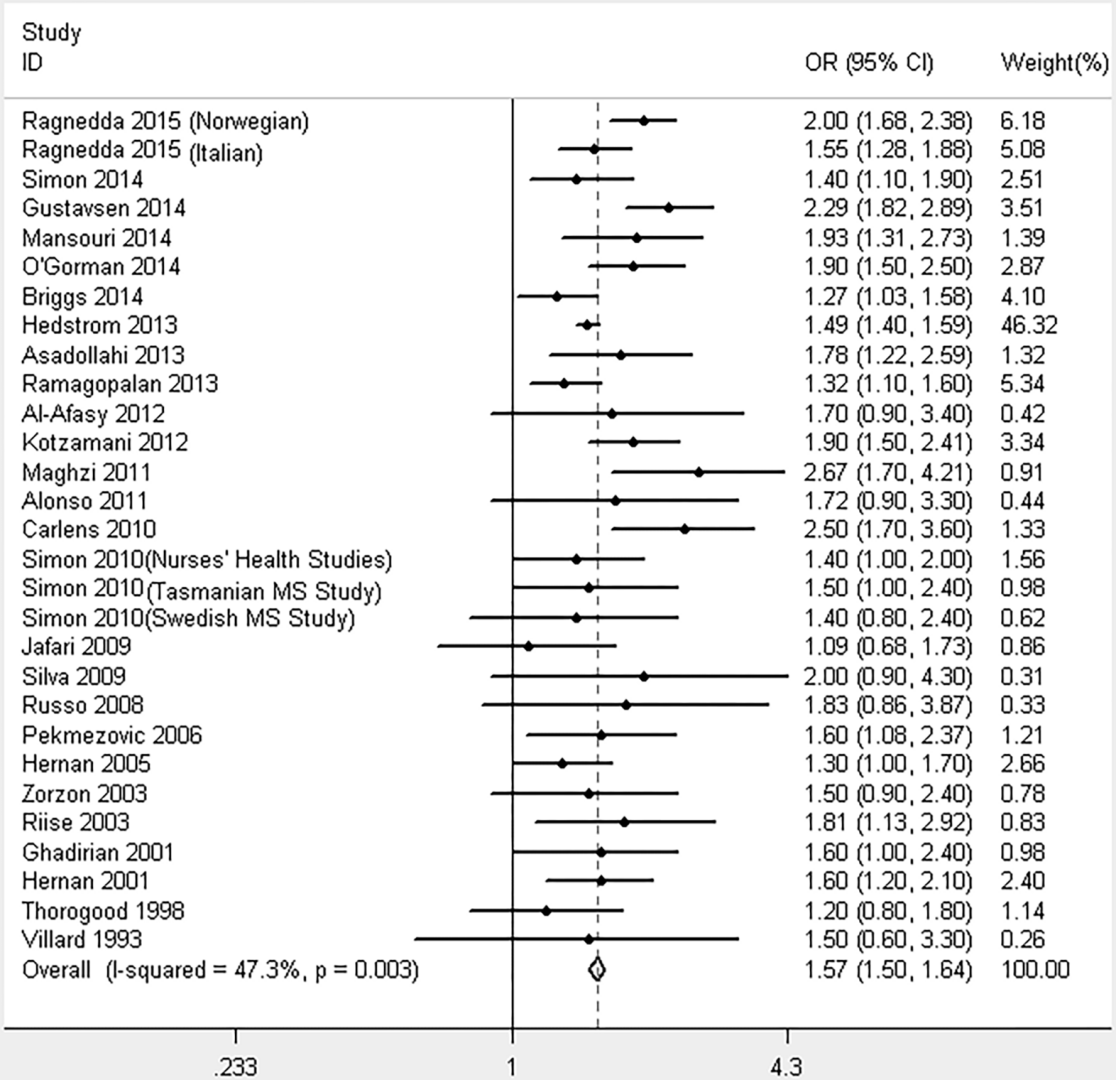
Forest plot of smoking and multiple sclerosis risk (non-conservative model).

The conservative model contained 24 studies that investigated the association between smoking and MS. Moderate heterogeneity was detected (*I*^2^ = 37.2%, *p* = 0.035). As described in Fig. 2, the pooled OR was 1.55 (95% CI [1.48–1.62], *p* < 0.001), indicating that ever-smoking increases the risk of MS by 55% compared with never-smoking individuals. When including all 29 studies in the non-conservative model, we obtained similar results (OR = 1.57, 95% CI [1.50–1.64], *p* < 0.001, heterogeneity: *I*^2^ = 47.3%, *p* = 0.003; Fig. 3). There were no significant differences among the subgroups based on study designs, diagnostic criteria, or the data collection methods; however, not adjusting for confounders may overestimate the risk between smoking and MS susceptibility (Table 2).

**Table 2:** Odds ratio and 95% confidence intervals for different subgroups of studies.

Subgroups	Number of studies	Odds ratio	95% CIs	*p*-value for comparison
Case-control	24	1.56	1.49–1.63	0.362
Cohort	5	1.70	1.42–2.03	
McDonald/ Poser criteria	16	1.70	1.52–1.90	0.124
Physician/self-reported/not reported	13	1.52	1.39–1.66	
Adjustment for covariates	15	1.51	1.43–1.59	0.005
No adjustment	14	1.74	1.60–1.89	
Self-administrated questionnaire	14	1.58	1.43–1.74	0.674
Face or telephone interview/not report	15	1.63	1.47–1.82	

### Different effects of genders and smoking habits

**Figure 4:**
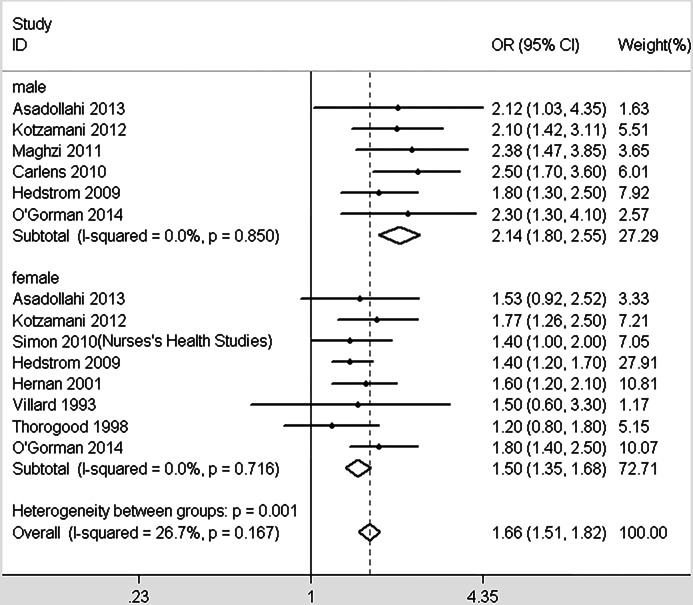
Forest plot of smoking and risk of multiple sclerosis in different genders.

**Figure 5:**
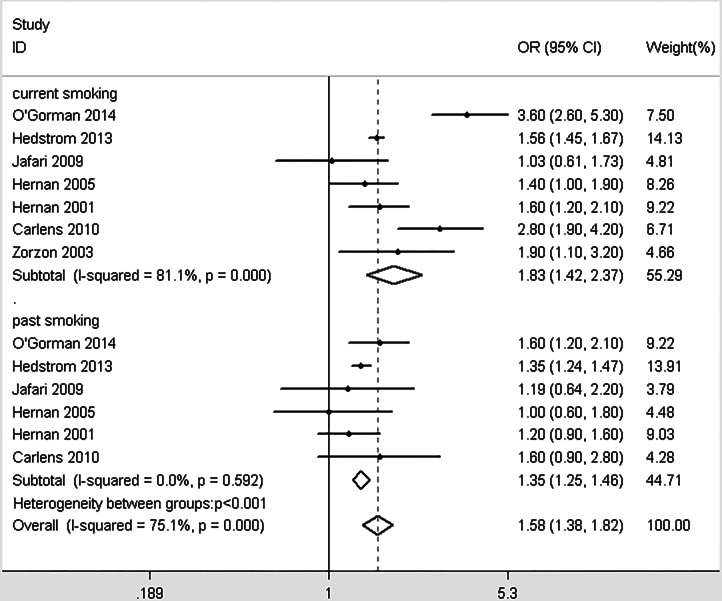
Forest plot of smoking and risk of multiple sclerosis in different smoking habits.

**Figure 6:**
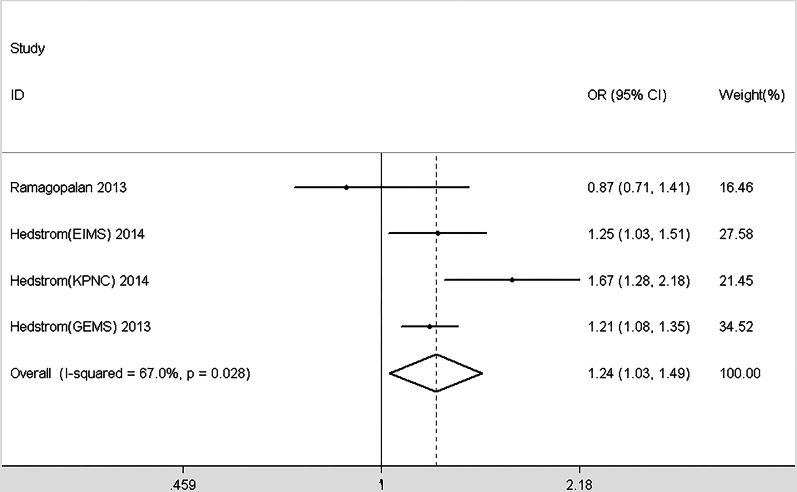
Forest plot of passive smoking and multiple sclerosis risk.

In total, 10 studies provided enough information to report the association between smoking and MS within genders (Asadollahi et al., 2013; Carlens et al., 2010; Hedstrom et al., 2009; Hernan, Olek & Ascherio, 2001; Kotzamani et al., 2012; Maghzi et al., 2011; O’Gorman et al., 2014; Simon et al., 2010; Thorogood & Hannaford, 1998; Villard-Mackintosh & Vessey, 1993). Significant differences were detected between different genders (*χ*^2^ = 11.21, *p* = 0.001, Fig. 4). Smoking in men is more dangerous than women. Similarly, we included 7 studies that provided data about the effects of different smoking habits on susceptibility to MS (Carlens et al., 2010; Hedstrom et al., 2013a; Hernan et al., 2005; Hernan, Olek & Ascherio, 2001; Jafari et al., 2009; O’Gorman et al., 2014; Zorzon et al., 2003). Being a current smoker increases the risk of MS by 83% risk compared with nonsmokers; past smoking increases the risk of MS by 58% compared with nonsmokers. Significant differences were detected between the effects of current and past smoking versus non-smokers (*χ*^2^ = 12.66, *p* < 0.001, Fig. 5). In order to explore the impact of passive smoking (active smokers were excluded) on the risk of MS, we identified 3 eligible articles containing four study populations (Hedstrom et al., 2014a; Hedstrom et al., 2013b; Ramagopalan et al., 2013). As described in Fig. 6, the pooled OR was 1.24 (95% CI [1.03–1.49], *p* = 0.028), indicating that exposure to passive smoking increases the risk of MS by 24% compared with unexposed individuals.

**Figure 7:**
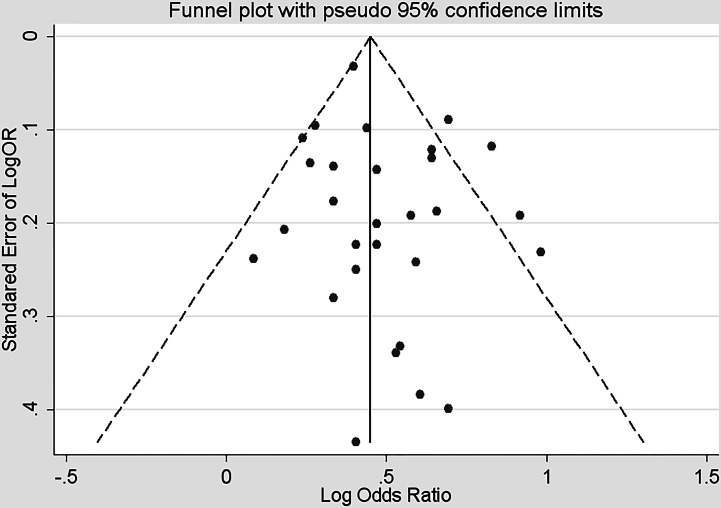
Funnel plot based on related risk for association between smoking and multiple sclerosis.

### Sensitivity analysis and publication bias

Figure 7 implied the funnel plot was symmetrical, suggesting no publication bias. The Begg rank correction test and Egger linear regression showed no asymmetry (Begg, *p* = 0.612; Egger, *p* = 0.204).

**Figure 8:**
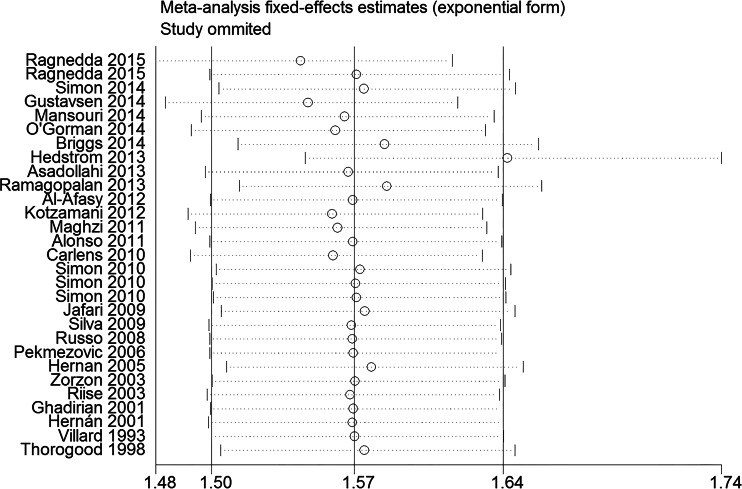
Forest plot of sensitivity analysis by removing each study in each turn.

Figure 8 showed the result of the sensitivity analysis by removing one study in each turn. This procedure showed that the study by Hedstrom in 2013 significantly impacted the main result. When switched from fixed effects model to random effects model, the OR changed from 1.57 (95% CI [1.50–1.64], *p* < 0.001) to 1.63 (95% CI [1.51–1.76], *p* < 0.001), suggesting that the result was robustness.

## Discussion

Our meta-analysis showed there was a strong association between smoking and MS susceptibility. Ever-smoking could increase the risk of MS by a more than 50% risk compared with never-smoking population. The non-conservative model obtained a similar result compared with the conservative model, suggesting a robustness of the results. The subgroup analyses showed that different study designs, diagnostic criteria and types of information resource had little impact on the relationship between smoking and MS susceptibility. However, inadequate adjustment may overestimate the risk between smoking and MS susceptibility. The sensitivity analysis showed the study by Hedstrom 2013 significantly impacted the main result. Therefore, we reviewed this article and found that it included 6,990 cases (no snuff use) and 8,279 controls (no snuff use) that constituted 46.32% of the entire meta-analysis. Male smokers were shown to have a higher risk of developing MS than female, but the exact number of cigarettes consumed by different genders per day due to different lifestyle habits was unavailable, so we were unable to draw a firm conclusion. Significant differences were detected between the effects of current and former smokers compared with non-smokers. Current smoking is more dangerous than past smoking, which informed individuals of the benefits of smoking cessation. Passive smoking is a risk factor for MS in non-smoking population. Smoke-free environment in public places and home is vital to people’s health.

Comparing with three former meta-analyses (Hawkes, 2007 (OR = 1.34), Handel 2011 (OR = 1.52), O’Gorman 2014 (OR = 1.51)), our study obtained a greater effect estimates between smoking and MS susceptibility (OR = 1.57) (Handel et al., 2011; Hawkes, 2007; O’Gorman & Broadley, 2014). Studies published from 2013 to 2015 accounted for 78.62% of the entire meta-analysis and reported higher effect estimates.

The etiology of MS is still unknown, and both genetic and environmental factors may contribute to this disease (Compston & Coles, 2008). MS is more common with the increasing latitude, except for some ethnic groups such as the New Zealand Moori (Pugliatti, Sotgiu & Rosati, 2002), Canada’s Inuit (Milo & Kahana, 2010) and inland Sicilians (Grimaldi et al., 2001); however, the reasons for these geographical distributions are still controversial (Milo & Kahana, 2010). Some people believe that a possible explanation could be that decreased exposure to sunlight results in decreased levels of vitamin D (Ascherio & Munger, 2007; Ascherio, Munger & Simon, 2010), while others believe that it is a consequence of the distribution of the northern European populations that had a high prevalence of MS (Milo & Kahana, 2010). Although MS is not considered to be a hereditary disease, the probability of MS is higher if there is a family history of the disease (Compston & Coles, 2002). Differences of specific genes in the human leukocyte antigen (HLA) system that serve as the major histocompatibility complex (MHC) may be associated with MS susceptibility (Compston & Coles, 2008).

The causal link between cigarette smoking and MS is still unclear (Jafari & Hintzen, 2011). There are more than 4,500 types of possible toxic substances, including nicotine and nitric oxide in cigarette smoke. Some nerve lesions, such as axonal degeneration, have been caused by exposure to nitric oxide (Scolding & Franklin, 1998; Smith, Kapoor & Felts, 1999). A study in Sweden showed the inhalation of non-nicotinic components of cigarette smoke are more influential than nicotine in the etiology of MS (Carlens et al., 2010). This finding suggests the real reason for the elevated risk of MS is the irritation of cigarette smoke in the lungs, triggering the pro-inflammatory effect of smoking via toll-like receptors (Mortaz et al., 2009; Pace et al., 2008). As a type of lymphocyte, T-cells enter the brain by destroying the blood–brain barrier in the inflammatory process. The T-cell recognized myelin as exogenous material and attacked it, causing the loss of myelin (Compston & Coles, 2008). Further damage of the blood–brain barrier will lead to a number of other effects, such as the activation of cytokines and modification of proteins that may break self-tolerance, resulting in autoimmune responses against antigens of the nervous system (Makrygiannakis et al., 2008).

Most of the studies included in this meta-analysis focus on the risk of MS between having ever smoked and never smoking; however, the exact dose of cigarette consumption as well as how these data were recorded vary from study to study (pack-years, per day etc.). Therefore, it is difficult to assess the association between the degree of MS susceptibility and the degree of cigarette consumption based on current studies.

## Conclusions

Our meta-analysis suggests that exposure to smoking is an important risk factor for MS. People would benefit from quitting smoking, and policymakers should pay attention to this association. Further research is needed to assess the dose–response effect between smoking and MS.

## Supplemental Information

10.7717/peerj.1797/supp-1 Supplemental Information 1 PRISMA checklist

10.7717/peerj.1797/supp-2 Supplemental Information 2 Raw data

## Additional Information and Declarations

### Data Availability

The following information was supplied regarding data availability: The research in this article did not generate any raw data.
